# "Good Is Up” Is Not Always Better: A Memory Advantage for Words in Metaphor-Incompatible Locations

**DOI:** 10.1371/journal.pone.0108269

**Published:** 2014-09-26

**Authors:** L. Elizabeth Crawford, Stephanie M. Cohn, Arnold B. Kim

**Affiliations:** Department of Psychology, University of Richmond, Richmond, Virginia, United States of America; Emory University, United States of America

## Abstract

Four experiments examined whether memory for positive and negative words depended on word location and vertical hand movements. Cognitive processing is known to be facilitated when valenced stimuli are presented in locations that are congruent with the GOOD is UP conceptual metaphor, relative to when they are presented in incongruent locations. In both free recall and recognition tasks, we find a memory advantage for words that had been studied in metaphor incongruent locations (positive down, negative up). This incongruity advantage depends on the location of words during encoding, but no evidence was found to suggest that other spatial associations, such as the vertical position of the hand at encoding or word location during retrieval, affect memory. The results indicate that metaphors, like schemas, categories, and stereotypes, can influence cognition in complex ways, producing variable outcomes across different tasks.

## Introduction

In keeping with the mind-as-computer metaphor, early work in cognitive psychology focused on information processing and paid little attention to the physical bodies that brains evolved to serve. Some canonical examples are the classic studies of memory for words or nonsense words in the absence of any meaningful context (e.g., [1], [2], [3]). Recently the growing trend toward an embodied, situated approach to cognition has shifted the field’s focus to cognition in the context of physical, bodily, and temporal constraints.

One thread of this approach is conceptual metaphor theory, which claims that we represent abstract ideas in terms of more concrete, physically embodied ones [4]. For example, in the GOOD is UP metaphor, the vertical dimension of space is used to conceptualize valenced states such as happiness, health, status, and morality. This is reflected in linguistic descriptions such as “being uplifted,” “feeling under the weather,” “climbing to the top of the profession,” or “falling from grace.” These uses of figurative language are thought to reflect an underlying cognitive principle: We use our understanding of verticality to conceptualize valence.

Recent empirical research has provided support for this view by showing that the link between valence and verticality influences cognitive processing (for reviews, see [5], [6]). Meier and Robinson [7] had participants evaluate a centrally located positive or negative word and then identify as quickly as possible a symbol that appeared above or below center. Participants were faster to shift attention upward when they had just evaluated a positive word and downward when they had evaluated a negative word, compared to the opposite pairing of valence and location. Thus, immediate, time-pressured processing was facilitated when information was presented in a manner that was congruent with the GOOD is UP metaphor, compared to when it was incongruent (see also [8], [9]). Similar facilitation effects have since been found for other affective metaphors, including GOOD is BRIGHT [10], GOOD is BIG [11], and GOOD is HIGH PITCHED [12].

Almost all experimental studies of conceptual metaphor focus on immediate, online judgments about presented stimuli and use reaction time as a dependent measure, and yet one of the hallmarks of human cognition may be our ability to deliberate about, plan for, and remember that which is not immediately present (cf., [13]). Rather than responding to our immediate physical environment, these forms of offline cognition require that we suppress information coming in from that environment in order to imagine alternatives. If metaphors, and embodiment more generally, play a powerful role in cognition, they would be expected to influence offline cognition as well. Here we investigate their role in memory.

Relatively few studies have moved beyond the here-and-now focus of reaction time measures to examine how conceptual metaphors affect memory. Testing memory for location, Crawford, Margolies, Drake and Murphy [14] found that positive stimuli are recalled as having appeared higher in space than comparably located negative stimuli. In a study of autobiographical memory, Casasanto and Dijkstra [15] found that people recalled more positive memories while they were moving marbles upward and more negative memories while moving marbles downward. Most relevant for the current study, Palma, Garrido, and Semin [16] included a surprise memory test in a study that asked participants to read behavioral traits to form an impression of either a childcare worker (positive stereotype) or a skinhead (negative stereotype). Participants remembered more descriptions that had been placed in metaphor-congruent than in metaphor-incongruent locations. The experiments reported here take a more direct approach to the question of how metaphors affect memory by examining it without the social goal of forming impressions of stereotyped individuals. Using two classic cognitive psychology paradigms, recognition and free-recall, we investigated whether memory for valenced words depends on whether they are presented in locations congruent or incongruent with the GOOD is UP metaphor.

Words convey their meanings regardless of their position on a page, and it is usually the relative ordering of the words and not their location in space that matters. Furthermore, in a task that asks people to study and later recall individually presented words, the location of the words is incidental and apparently irrelevant to the task. From this point of view, spatial position would not be expected to have any impact.

However there is empirical evidence that spatial and lexical processing are interrelated. In the spatial Stroop paradigm, participants identify spatial words such as “above” or “below” as they appear in various spatial locations, and reaction times are slower when the word meaning and location are incongruous (e.g., [17]). Spatial-lexical interactions are also found for words that are not spatial terms, but refer to objects that have canonical spatial relations. Zwaan and Yaxley [18], showed pairs of words such as “attic” and “basement,” and asked participants to judge whether the words were semantically related. Judgments were found to be faster when the words’ spatial locations were congruent with their referents’ canonical locations (i.e., “attic” shown above “basement”) than when they were incongruent. As people access the meanings of words, they seem to spontaneously activate associated spatial information.

This connection between lexical and spatial processing may have implications for memory. The facilitated processing that has been observed when information is presented in a metaphor-congruent manner may produce downstream advantages for memory (as in [16]). Such an outcome could stem from several mechanisms. For example, if metaphor-congruent information is easier to process, this may free up cognitive resources that could be devoted to more elaborate encoding. In addition, words in metaphor congruent as opposed to incongruent locations may produce a stronger affective response because the location reinforces the valence of the word, and content that is more emotionally charged is known to be better remembered [19]. Thus we might expect memory to show the same metaphor-compatibility advantage that reaction time studies have shown.

However, there is reason to be cautious about using reaction time advantages to predict memory advantages, as memory can be well served by more difficult processing. For example, words that are immediately followed by an interfering perceptual mask are harder to read but better remembered than unmasked words [20], [21]. In addition, when perceptual dysfluency is increased by printing items in difficult-to-read fonts, memory for those items is enhanced [22]. In addition, research on stereotype violation has shown that unexpected information is sometimes better remembered than information that fits people’s prior expectations [23], and in the categorization literature, a similar effect is observed for stimuli that violate a categorization rule [24]. Words presented in metaphor-incongruent locations may present similar processing challenges, which could lead them to be better remembered than those presented in metaphor-congruent locations.

## Experiment 1: Free Recall

### Methods

#### Ethics Statement

All experiments reported in this paper were conducted with approval from the University of Richmond Institutional Review Board (1565) that was convened on 11/5/13 by Dr. Kirk Jonas, Chair. Written informed consent was obtained for each participant.

#### Participants

Eighteen University of Richmond undergraduates, all at least 18 years old, participated in exchange for partial course credit.

#### Materials and Procedure

Twenty positive and twenty negative words were selected from the Affective Norms for English Words (ANEW) [25], which have been normed on dimensions of valence and arousal. Words were selected so that the positive and negative words would be comparable in arousal and in extremity of valence. (On a 9-point scale, the selected positive and negative words had mean arousal scores of 4.32 and 4.47, respectively, which do not differ significantly, *t*(38) = 1.01, *p* = .32. Their mean valence scores were 7.17 and 2.88, which do not differ significantly in their deviation from the midpoint of the scale, *t*(38) = −.28, *p* = .78). Words referring to objects with canonical locations (e.g., “sun”) were avoided. After informed consent procedures, participants were seated about 20 inches from a 17-inch computer monitor and told that they would read a series of words on which they would later be tested. Each word was shown individually in black 28-point font on a white background, for 1000 ms with a 500-ms blank screen in between presentations. Words were randomly ordered and randomly assigned to appear in the center of the top half or bottom half of the display. After all words had been presented, participants were given a blank text document and asked to type in as many words as they could remember at their own pace.

### Results and Discussion

Memory intrusions were rare (∼2% of responses) and were not analyzed. A response was coded as correctly recalled if the typed word matched the presented word. There were seven trials on which participants responded with different spellings or forms of the words that had been shown, such as writing “crime” when the presented word was “criminal.” These were not counted among the correct responses. An independent coder recoded 20% of the data and agreed with the first coder on all trials.

**Figure 1:**
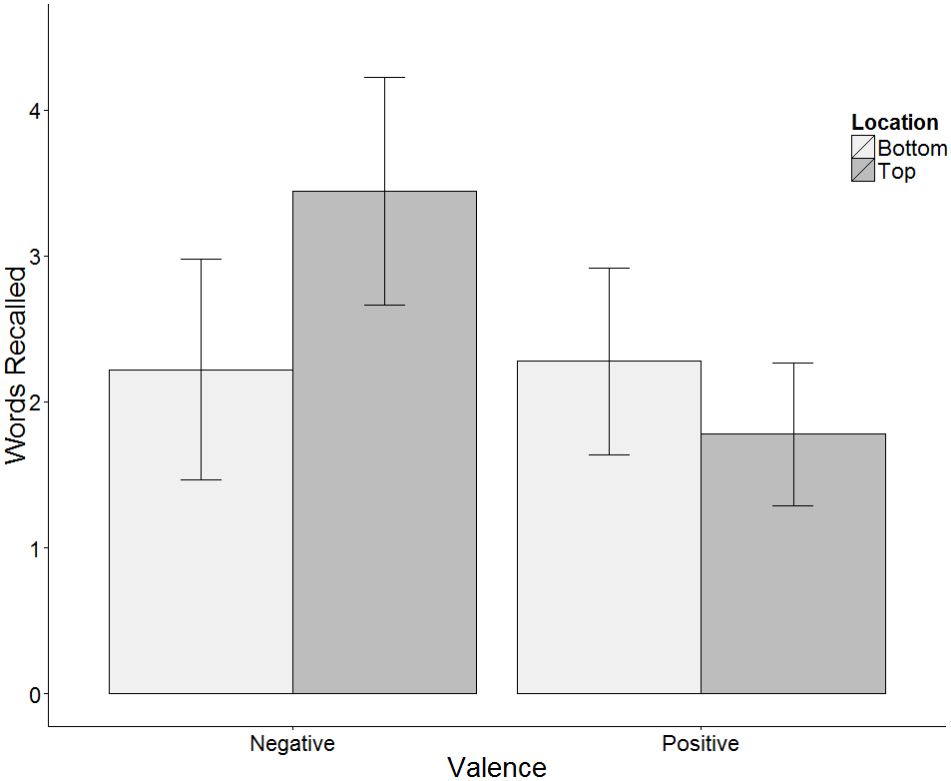
Mean number of words recalled by valence and location. Experiment 1.

There was a significant interaction between valence and location (*F*(1, 17) = 5.69, *p*<.05, *η*_p_^2^ = .25), indicating that the effect of valence on recall depended on where stimuli were located. Cell means are depicted in Figure 1. Although location comparisons within valence did not reach significance, the pattern of the interaction suggests that stimuli that were incongruent with the GOOD is UP metaphor were better recalled than those that were congruent (Negatives on top: *M* = 3.44 (*SE* = .40), Negatives on bottom: *M* = 2.22 (.38), *t*(17) = 2.05, *p* = .056. Positives on top, *M* = 1.78 (.25), Positive on bottom: *M = *2.28 (.32), *t*(17) =  −1.29, *ns*.) The association between valence and verticality influences recall, but unlike previous studies, here it produces an advantage for information that was incongruent with the GOOD is UP metaphor.

In addition, there was a main effect of valence, with more negative than positive words recalled (*F*(1,17) = 7.2, *p*<.05, *η*_p_^2^ = .30). There was no main effect of location (*F*(1,17) = 1.05, *ns*).

We next examined whether the effect would generalize to recognition memory. An advantage of the recognition memory testing paradigm is that it can be used to manipulate vertical position at encoding and at retrieval, allowing us to examine the stage of processing at which such effects occur.

## Experiment 2: Recognition Memory, Location Manipulated at Encoding

### Methods

#### Participants

Thirty-four University of Richmond undergraduates, all at least 18 years old, participated in exchange for partial course credit.

#### Materials and Procedure

The study phase was identical to Experiment 1 except that participants viewed 41 positive and 41 negative words from the ANEW. Positive and negative stimulus words had mean arousal sores of 4.44 and 4.47, respectively, which do not differ significantly, *t*(80), = .27, *p* = .79. Their valence means were 6.88 and 2.97, which do not differ significantly in their deviation from the midpoint of the scale, *t*(80) = .92, *p* = .36. Immediately after studying the words, participants were given a recognition memory test in which they were shown the same words along with an additional 41 positive and 41 negative foils, which were selected from the same database and had comparable valence and arousal ratings as the test words. Among foils, the positive and negative words had mean arousal scores of 4.43 and 4.46, respectively, which do not differ significantly, *t*(80) = .46, *p* = .64. Their mean valence scores were 6.86 and 3.01, which do not differ significantly in their deviation from the midpoint of the scale, *t*(80) = .86, *p* = .39. All test words were shown in the center of the screen and participants pressed labeled keys to indicate whether each was “new” or “old” as quickly as possible. The word remained on the screen until one of the keys was pressed, after which there was a blank screen for 500 ms, followed by the next test word.

### Results and Discussion

Data from one participant was culled for poor accuracy. In addition, responses that occurred within 200 ms of test word onset were culled.

**Figure 2:**
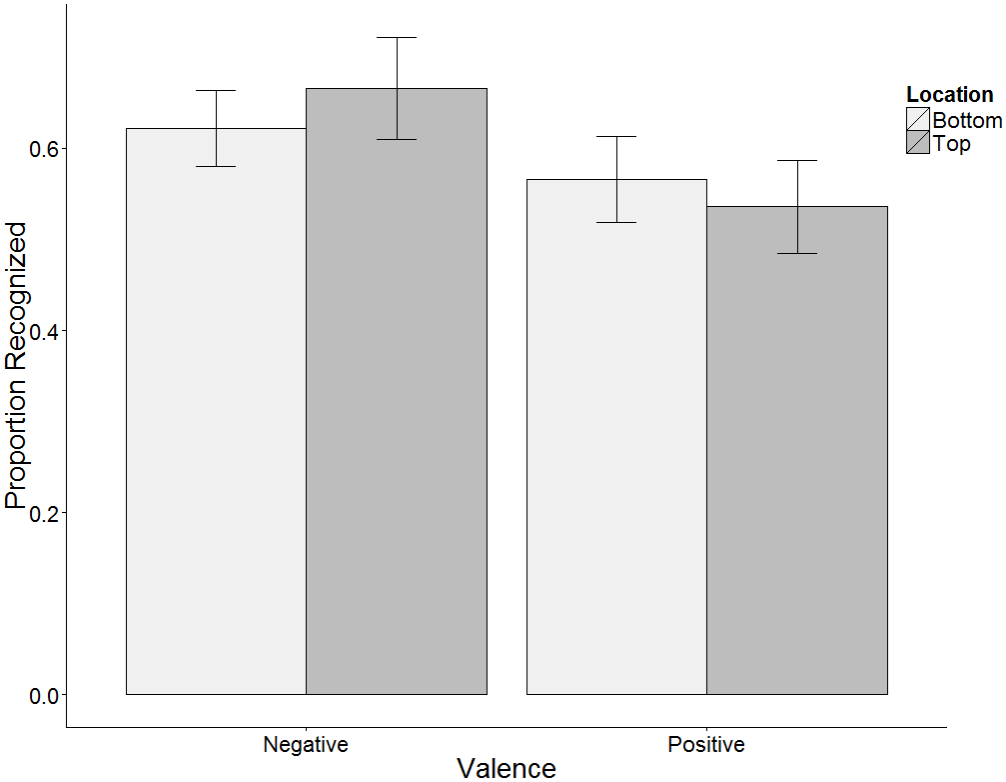
Mean proportion of words recognized by valence and location. Experiment 2.

For words that had been shown during the study phase (i.e., words for which location had been varied), there was a significant interaction between valence and location (*F*(1,32) = 4.48, *p*<.05, *η*_p_^2^ = .12). As in the free recall experiment, negative stimuli that had been studied in the top half of the screen were somewhat better recognized than those that had been studied in the bottom half (Negatives on top: *M* proportion correctly recognized = .68 (03), Negatives on bottom: *M* = .63 (.02), *t*(32) = 1.94, *p* = .061). Among positive stimuli, recognition did not depend on location (Positive on top: *M* = .55 (02), Positive on bottom: *M* = .85 (.02), *t*(32) = −1.34, *ns,* see Figure 2). It is not possible to use signal detection measures such as *d’* to examine whether location affects sensitivity because location was only manipulated at study, and thus cannot contribute to false alarms. Because information about different locations was not available at the time of response, any effects of it must be attributable to memory and cannot be readily explained by a response bias. Thus the results are consistent with those of Experiment 1 in showing that the effect of studied location on memory for words depends on word valence, and that the advantage is for words that were studied in locations incongruent with the GOOD is UP metaphor.

The analysis of variance revealed no main effect for location (*F*(1,32) = .09, *p = .77, η*_p_^2^ = .003), but did show a main effect of valence such that previously shown negative words were recognized more often than positive ones (Negative proportion recalled: *M* = .65 (.02), Positive: *M* = .57 (.02), *F*(1,32) = 32, *p*<.001, *η*_p_^2^ = .50). However an additional analysis using all stimuli (i.e., both previously shown and foils) showed no difference in sensitivity (*d’*) for positive and negative words (Negative *d’ M* = 1.14 (.09), Positive *d’ M* = 1.15 (.11), *t*(32) = .09, *ns*). Thus there appears to be a bias to respond “old” to negative stimuli regardless of whether they were shown previously.

## Experiment 3: Recognition Memory, Location Manipulated at Test

### Methods

#### Participants

Thirty University of Richmond undergraduates, all at least 18 years old, participated in exchange for partial course credit.

#### Materials and Procedure

The materials and procedure were the same as in Experiment 2, except for the manipulation of location. During the study phase, all words appeared in the center of the screen, whereas during the test phase, the words were randomly assigned to appear in the top or bottom of the display.

### Results and Discussion

Data from three participants was culled due to poor average accuracy. Sensitivity (*d’*) scores were submitted to an ANOVA with test location and valence as within-subjects factors. Unlike the first two experiments, there was no interaction between these variables (*F*(1, 26) = .95, *p* = .34,, *η*_p_^2^ = .035). When location is varied at the test stage of a recognition memory test, there is no evidence that its effect depends on the valence of words. The effects appear to be specific to the encoding stage.

Location did have a main effect on sensitivity, as sensitivity was greater when the test words appeared in the top half of space than in the bottom half (Top *d’ M* = 1.41 (.09), Bottom *d’ M* = 1.22 (.074); *F*(1,26) = 4.43, *p*<.05, *η*_p_^2^ = .15). Consistent with Experiment 1 (but not Experiment 2), there was there was also a main effect of valence, showing greater sensitivity for negative than for positive stimuli. (Negative *d’ M* = 1.45 (.08), Positive d’ *M* = 1.19 (.08), *F*(1,26) = 13.16, *p*<.01, *η*_p_^2^ = .34).

## Experiment 4: Recall, Hand Position Manipulated at Encoding

The previous experiments indicate that memory for valenced words is affected by whether they were initially encoded in metaphor congruent or incongruent spatial positions. Experiment 4 sought to determine if this effect was specific to word location, or whether spatially directed physical actions would produce similar effects. Here we use a free recall task to examine whether holding a hand up or down during encoding influences memory of metaphor congruent word stimuli.

### Methods

#### Participants

Forty University of Richmond undergraduates, all at least 18 years old, participated in exchange for partial course credit.

#### Materials and Procedure

Twenty four positive and 24 negative words taken from the ANEW were shown centrally on a computer screen. Within each valence category, half of the words appeared in a purple font and half in maroon. Participants stood in front of a bookshelf with three boxes on it, positioned at 37.5, 53, and 67.5 centimeters from the floor. Participants began each trial with one of their hands on the middle box and were told to hold it there until a word appeared. When the word was shown, they were told to shift their hand to the upper box or lower box, depending on the color of the word, with purple indicating up and maroon down. Participants held their hand on the box until the word disappeared (a total of 3000 ms) and then returned their hand to the middle box and awaited the next trial. Participants were encouraged to switch hands if their arms became tired. The order of trials was randomized and two different random orders were used so that words assigned to each color from one order were assigned to the other color in the second order. Participants performed the task while facing the bookshelf and standing shoeless on an electrically grounded piece of sheet metal. The boxes were covered in tinfoil and wired to a MaKey MaKey (makeymakey.com) device that enabled us to monitor when participants were making contact with each of the boxes. Immediately after the final word was shown, participants were given a clipboard and asked to write down as many words as they could remember.

### Results and Discussion

Responses were coded as in Experiment 1. The results showed no significant interaction between valence and the location of the hand during encoding (*F*(1,39) = .721, *p* = .401, *η*_p_^2^ = .018) and no main effect of hand position (*F*(1, 39) = .283, p = .598, *η*_p_^2^ = .007). Thus while Experiment 1 suggested that the metaphor-congruence of word location during encoding affects subsequent recall, this effect does not appear to generalize to the metaphor-congruence of hand position during encoding. This suggests that the effect is grounded in external space rather than the body itself. Consistent with the results of Experiments 1 and 3, experiment 4 found a significant effect of word valence, as participants recalled more negative than positive words (Negatives, *M* = 4.13 (*SE* = .28); Positives, *M* = 3.33(.29), *F*(1, 30) = 5.929, *p*<.05, *η*_p_^2^ = .132).

## Discussion

Memory for valenced words depends on the vertical position in which they were studied. For negative, but not positive, words, those that had appeared in the upper half of a screen were better remembered than those that had appeared in the lower half. There appears to be a memory advantage for words studied in locations that were incongruent with the GOOD is UP metaphor, at least for negative stimuli, and this effect generalizes across free recall and recognition memory tests.

An additional recognition test showed that while location of words during initial study has this effect, the location of test words during recognition does not. This suggests that the results stem from a stronger memory trace of the studied metaphor-incongruent stimuli, rather than facilitation at their retrieval. In addition, the incongruency advantage only occurred when the spatial position of the word itself was manipulated; varying participants’ hand locations at encoding did not affect memory.

Although the words used here were chosen to be comparable on extremity of valence and arousal dimensions, we find two notable differences between positive and negative stimuli. First, in three of the four experiments, negative words were better remembered than positive words. Previous studies on memory for valenced stimuli have produced mixed results, with some showing a positivity advantage [26]. It is not clear why negative information was better remembered here, but an intriguing possibility is that the added spatial component was a factor. Spatial working memory is known to be selectively enhanced by negative mood [27], but it is not known if increasing the spatial demands of a task influences emotion or the processing of affectively charged content. Second, the metaphor incongruency advantage appeared to be stronger for negative than for positive stimuli. This may reflect asymmetries in affective processing, as people are known to react more strongly to negative than to positive stimuli [28], possibly leading to greater activation of the metaphorically associated vertical position.

These findings contrast with previous empirical studies of the GOOD is UP metaphor, which have shown facilitated performance when stimuli or actions are metaphor-congruent rather than incongruent [15], [16]. It should be noted that because the procedures and measures used here differed substantially from those in prior work, there are many possible reasons for the differences in findings. Casasanto and Dijkstra [15] investigated autobiographical event memories, rather than having participants study stimuli in the laboratory, and they measured valence and retrieval time rather than recall or recognition accuracy. More similar to the present work is the recent study by Palma et al. [16], which showed a congruency advantage in memory for behavioral descriptions of an individual who belonged to either a positive or negative social category. Perhaps the most important difference between that and the current work is that Palma et al.’s participants viewed descriptions of a single stereotyped individual of whom they were to form an impression. It may be the case that this more complex social judgment led participants to adopt a processing style that favored information that was consistent with prior expectations. In the context of this prior work, the incongruency advantage found here suggests that metaphors, like schemas, stereotypes, and categories, can influence cognition in complex ways, producing variable outcomes across different tasks.
